# Haploidentical versus HLA-matched sibling transplantation for refractory acute leukemia undergoing sequential intensified conditioning followed by DLI: an analysis from two prospective data

**DOI:** 10.1186/s13045-020-00859-5

**Published:** 2020-03-12

**Authors:** Sijian Yu, Fen Huang, Zhiping Fan, Li Xuan, Danian Nie, Yajing Xu, Ting Yang, Shunqing Wang, Zujun Jiang, Na Xu, Ren Lin, Jieyu Ye, Dongjun Lin, Jing Sun, Xiaojun Huang, Yu Wang, Qifa Liu

**Affiliations:** 1 Department of Hematology, Nanfang Hospital, Southern Medical University, No.1838 North Guangzhou Avenue, Guangzhou, 510515 China; 2 Department of Hematology, Peking University People’s Hospital, Peking University Institute of Hematology, No.11 South Street of Xizhimen, Xicheng District, Beijing, 100044 China; 3 Department of Hematology, Sun Yat-Sen Memorial Hospital, Sun Yat-Sen University, No.107 Yanjiang West Road, Guangzhou, 510120 China; 4 Department of Hematology, Xiangya Hospital, Central South University, No.87 Xiangya Road, Changsha, 410008 China; 5 Department of Hematology, Fujian Institute of Hematology, Fujian Medical University Union Hospital, No.29 Xinquan Road, Fuzhou, 350001 China; 6 Department of Hematology, Guangzhou First People’s Hospital, No.1 Fupan Road, Guangzhou, 510180 China; 7 Department of Hematology, Guangzhou General Hospital of Guangzhou Military Command, No.111 Liuhua Road, Guangzhou, 510010 China; 8 Department of Hematology, the Third Affiliated Hospital, Sun Yat-Sen University, No.600 Tianhe Road, Guangzhou, 510000 China; 9 Department of Hematology, Nanhai Hospital, Southern Medical University, Foshan, China

**Keywords:** HLA-matched sibling, Haploidentical, Refractory acute leukemia, Similar survival, Transplantation

## Abstract

**Background:**

Compared with HLA-matched sibling donor (MSD) transplant, the outcomes of haploidentical donor (HID) transplant for refractory acute leukemia need to be further explored. In this study, we compared the outcomes of HID with MSD for refractory acute leukemia.

**Patients and methods:**

This study population came from two prospective multicenter trials (NCT01883180, NCT02673008). Two hundred and seventy-eight patients with refractory acute leukemia were enrolled in this study, including 119 in HID group and 132 in MSD group. Sequential intensified conditioning was employed in all patients, and donor lymphocyte infusion (DLI) was administered in patients in the absence of active GVHD and according to minimal residual disease (MRD) from day + 60 post-transplantation for preventing relapse.

**Results:**

The complete remission of leukemia by day + 30 post-transplant were 94% and 93%, respectively, in HID and MSD groups (*p* = .802). The 1-year incidence of grades II–IV acute GVHD was 62% and 54% (*p* = .025), and 3-year incidence of chronic GVHD was 55% and 55% (*p* = .789), respectively, in two groups. HID transplant had lower incidence of first episode of MRD positivity and relapse than MSD transplant (28% vs 45%, *p* = .006; 26% vs 38%, *p* = .034). There was higher infection-related mortality in HID than MSD (8% vs 2%, *p* = .049) within the first 100 days’ post-transplant. The 5-year overall survival was 46% and 42% (*p* = .832), respectively; the 5-year disease-free survival was 43% and 39% (*p* = .665), in HID and MSD groups, respectively.

**Conclusions:**

HID transplant has lower relapse, but higher infection-related mortality and similar survival rates in refractory acute leukemia by the strategy of sequential intensified conditioning followed by DLI compared with MSD transplant.

## Introduction

So far, allogeneic hematopoietic stem cell transplantation(allo-HSCT)remains the most effective way to cure refractory leukemia [1–4] and undergoing allo-HSCT promptly is essential for them. Recently, some studies showed that intensified conditioning followed by allo-HSCT could achieve acceptable outcomes for refractory leukemia [5–9]. In our transplant center, a strategy of sequential intensified conditioning followed by donor lymphocyte infusion (DLI) was implemented since 2009, and encouraging outcomes were reported previously [10].

Quick access to appropriate donors is one of the key elements to the success of transplantation for refractory leukemia. As it is known, only 25–30% patients can get a HLA-matched sibling donor (MSD), and most patients cannot wait to search for a suitably matched unrelated donor if it is not already available. With improvements having been made in haploidentical donor (HID) transplant strategies, some studies showed that transplant outcomes of HID were similar to MSD in acute leukemia [11–14], but data comparing HID with MSD for refractory leukemia are quite limited [15, 16]. A recent retrospective analysis from EBMT [16] showed that HID transplant was associated with inferior survival rates and higher non-relapse mortality (NRM). However, the study population was very heterogeneous in terms of conditioning regimen, graft versus host disease (GVHD) prophylaxis, intervention strategy post-transplantation, and so on. Besides, some studies found a lower relapse incidence (RI) in HID than MSD transplant for high risk leukemia, suggesting superior graft versus leukemia (GVL) in HID [17–19].

In this study, we analyzed data from two prospective multicenter trials, to investigate the transplant outcomes of HID versus MSD for refractory acute leukemia by using our transplant strategy of sequential intensified conditioning followed by DLI [10].

## Patients and methods

This study population came from two prospective multicenter trials (NCT01883180, NCT02673008). Patients undergoing allo-HSCT between June 2013 and December 2017 were enrolled in this study if they met the following criteria: (1) refractory acute leukemia, (2) no complete remission (no-CR) at transplant, (3) using HID or MSD as donors, and (4) first allo-HSCT. Patients with FLT3-ITD and BCR/ABL were excluded because they received sorafenib or tyrosine kinase inhibitors post-transplant. The study was performed in accordance with the modified Helsinki Declaration, and the protocol was approved by our ethical review boards before study initiation. Informed consent was obtained from the donors and recipients.

### Conditioning and transplants

The sequential intensified conditioning regimen was used in all patients, including fludarabine (Flu) 30 mg/m^2^/day and cytarabine (Ara-C) 2 g/m^2^/day from days − 10 to − 6, 4.5 Gy of total body irradiation (TBI) on days − 5 and − 4, cyclophosphamide (CY) 60 mg/kg/day, and etoposide (VP-16) 15 mg/kg/day on days − 3 and − 2 [7]. As for donor selection, MSD (6/6 matching HLA-A, B, and DR loci) was the first choice. If MSD and a suitably matched unrelated donor (> 8 of 10 matching HLA-A, B,C,DR, and DQ loci) were unavailable, patients would be transplanted with HID [4].HID patients were transplanted with a combination of bone marrow (BM) and peripheral blood stem cell (PBSC) grafts, whereas MSD patients received PBSC grafts. Ciclosporin A (CsA) + methotrexate (MTX) were administered in patients undergoing MSD transplants for GVHD prophylaxis. CsA + MTX + antithymocyte globulin (ATG) and mycophenolate were used in patients receiving HID [10].The total dose of ATG (rabbit anti-human thymocyte immunoglobulin, ImtixSangstat, Lyon, France) was randomly assigned as 7.5 mg/kg from days − 3 to − 1 or 10.0 mg/kg from days − 4 to − 1 in one trial (NCT01883180), and just 10.0 mg/kg from days − 4 to − 1 for all patients in another trial (NCT02673008).

### Surveillance and intervention for relapse

BM samples analyzed at 1, 2, 3, 4, 6, 8, 10, and 12 months within 1 year post-transplantation, then at 3-month intervals from the 13th to 24th month, and 4-month intervals from the 25th to 36th month for the monitoring of morphology and minimal residual disease (MRD). If MRD was positive, the test was repeated in 1 week. Eight-color multiparameter flow cytometry (MFC) and quantitative PCR (qPCR) were used for the detection of MRD as previously described [20–22].For MFC method, positive MRD was considered when a cluster of more than 25 cells with leukemia-associated immunophenotypes (LAIP) and SSC characteristics identified in all plots of interest and carrying at least two LAIP markers identified at diagnosis that was observed. For those without LAIP markers at diagnosis, MRD was identified as a cell population showing deviation from the normal patterns of antigen expression seen on specific cell lineages at specific stages of maturation compared with either normal or regenerating marrow. A lower limit of detection of 0.01% was targeted. When abnormal cells were identified, the cells were quantified as a percentage of the total CD45 white cell events. Any measurable level of MRD was considered positive. Also, leukemia-related specific genes, including NPM1, RUNX1-RUNX1T1, and CBFβ/MYH11 were detected by qPCR in AML. The cutoff value was 0.001%. Subjects were defined as MRD+ if they had 2 consecutive positive results using FCM or PCR or were both positive in a single sample.

The prevention of leukemic relapse included early tapering of immunosuppressant, prophylactic, and preemptive therapies according to our previous literatures [10, 22]. CsA was withdrawn by 10%/week in patients without acute GVHD (aGVHD) by day + 30 post-transplantation. Prophylactic DLI was given in patients once on day + 90 post-transplantation when donor lymphocytes were available without active aGVHD [10]. Preemptive DLI was conducted in patients with MRD+ post-transplants (post-MRD+) without active GVHD from day + 60, which was given monthly until GVHD occurred or MRD became negative or for a total of 4 times. Donor lymphocytes were obtained from G-CSF mobilized peripheral blood [10, 23]. The CD3^+^ T cells count for per DLI (pDLI) was 3 × 10^7^/kg. Short-term immunosuppressive agents were used for prevention of GVHD after DLI [23]. For post-MRD+ AML patients with active GVHD, preemptive decitabine with a dose of 20 mg/m^2^/day for 5 consecutive days was administered monthly until MRD turned negative or hematological relapse or for a total of 4 times.

### Infection prophylaxis

Infection prophylaxis was performed as previously described [24, 25]. Oral sulfamethoxazole and norfloxacin were used in all cases. The EBV− and CMV-DNA loads in the blood were measured regularly by real-time qPCR. EBV-DNA or CMV-DNA was considered positive when the copies exceeded 500 copies/ml. Preemptive therapy was given to the patients with EBV or CMV-DNA-emia [25]. Antifungal agents were administered 5 days before transplantation. Oral fluconazole (0.3 g/day) was used for up to + 60 days post-transplantation in patients with no history of invasive fungal infection (IFI); for patients with a history of IFI, antifungal agents for secondary prophylaxis based on response to initial antifungal therapy were used for up to + 90 days post-transplantation [26].

### Evaluation endpoints and definitions

The primary endpoint included RI and overall survival (OS). Secondary endpoints included engraftment, disease response, GVHD, infections, NRM, disease-free survival (DFS), GVHD-free, and relapse-free survival (GRFS). Assessments of engraftment and chimerism were previously described in detail [7]. Relapse was defined by morphologic evidence in the peripheral blood, marrow, or extramedullary sites. On days 0 and + 30 post- transplantation, disease response was assessed by BM aspiration. CR was defined as< 5% blasts with no evidence of dysplasia in the BM and no manifestations of leukemia outside the hematopoietic system. Partial remission (PR) was defined as 5–20% blasts with or without extramedullary leukemia. Non-remission (NR) was defined as a failure to obtain CR. NRM was estimated as death without evidence of leukemia recurrence. DFS was defined as survival in continuous complete remission without relapse. GRFS events were defined as grades III–IV acute GVHD (aGVHD), chronic GVHD (cGVHD) requiring systemic immunosuppressive treatment, leukemia relapse, or death from any cause during follow-up after allo-HSCT [27]. aGVHD and cGVHD were graded according to the literatures [28, 29]. CMV-associated disease was defined by the presence of clinical symptoms or signs of end organ disease, combined with the evidence of CMV infection in a tissue biopsy specimen. EBV-associated diseases were classified into EBV-associated post-transplant lymphoproliferative diseases and EBV-associated other diseases [25]. Acute leukemia, including acute myeloid leukemia (AML), acute lymphoblastic leukemia (ALL), and acute leukemia of ambiguous lineage (ALAL), was defined according to World Health Organization guideline [30]. Refractory acute leukemia was defined as primary induction failure (PIF) after two or more cycles of chemotherapy or relapse refractory to salvage chemotherapy [7, 16].Genetics, including cytogenetics and molecular genetics, was defined as favorable, intermediate, and poor-risk in acute myeloid leukemia (AML) [31] and favorable and poor-risk in acute lymphoblastic leukemia (ALL) according to the National Comprehensive Cancer Network guideline [32]. Prophylactic and preemptive therapies were defined as interventions for MRD− and MRD+ patients without hematologic relapse, respectively.

### Statistical analysis

Analysis was performed on June 30, 2019. Variables related to patients, disease, and transplant characteristics between the two groups were compared using Fisher’s exact test for categorical variables and Mann-Whitney *U* tests for continuous variables. Numerical variables were analyzed as categories based on their values being below or above the median of the entire cohort. DFS, OS, and GRFS were calculated using the Kaplan-Meier method and compared by the log-rank test. Cumulative incidences were estimated for engraftment, GVHD, relapse, NRM, and infections to accommodate competing risks. Competing risk for engraftment was death without engraftment, competing risks for GVHD included death without GVHD and relapse, competing risks for infections included death without infections and relapse, relapse was a competing risk for NRM, and NRM was a competing risk for relapse. A cox proportional hazards model was used for analysis of risk factors for time-to-event variables. Fine and Gray model was used for analysis of endpoints involving competing risks [33].The following variables were included in the univariate analysis: donor type, gender, age, underlying diseases, genetics, white blood cell count at diagnosis, BM blasts pre/post-conditioning, MRD post-transplant, DLI, aGVHD, and cGVHD. Only variables with *p* < 0.10 were included in the multivariate analysis. *P* values of less than 0.05 were considered statistically significant. The Stata SE 12.0 (StataCorp LP) and R version 3.4.3 (R Development Core Team, Vienna, Austria) were used for all data analysis.

## Results

### Patients and transplant characteristics

**Fig 1:**
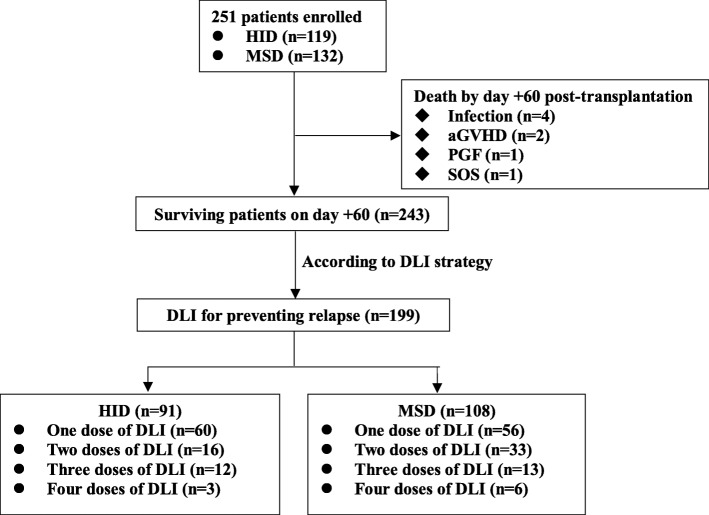
Flow diagram

There were 251 patients enrolled in this study, including 119 in HID group and 132 in MSD group (Fig. 1). The median age was 29 (range 14–56) years. Primary diseases included AML (*n* = 111), ALL (*n* = 115), and ALAL (*n* = 25). Patient and transplant characteristics are shown in Table 1. There was no significant difference between two groups in terms of baseline factors in Table 1 (*p* > .050).

**Table 1:** Patients and transplant characteristics

Characteristics	HID	MSD	*p*
No. of patients	119	132	–
Median age, years (range)	28 (14–54)	33 (16–56)	.127
Sex, no. (%)			.448
Male/female	65 (54.6)/54 (45.4)	65 (49.2)/67 (50.8)	
Underlying diseases, (%)			.731
AML	54 (45.4)	57 (43.2)	
ALL	55 (46.2)	60 (45.4)	
ALAL	10 (8.4)	15 (11.4)	
Disease stage at transplants, (%)			.447
Primary induction failure	58 (48.7)	57 (43.2)	
Relapse refractory	61 (51.3)	75 (56.8)	
WBC count at diagnosis, n (%)			.615
≥ 30,000 per mm^3^	57 (47.9)	59 (44.7)	
< 30,000 per mm^3^	62 (52.1)	73 (55.3)	
Median BM blasts before conditioning (range)	33% (7.0–96.0%)	31% (8.0–98.0%)	.602
Genetics, (%)			.527
Poor	65 (54.6)	66 (50.0)	
Intermediate	34 (28.6)	45 (34.1)	
Favorable	5 (4.2)	8 (6.1)	
Unknown	15 (12.6)	13 (9.8)	
Graft no. (%)			-
BM + PBSC	119 (100)	3 (2.3)	
PBSC	0 (0)	129 (97.7)	
Median MNCs, 10^8^/kg (range)	7.36 (3.76–12.80)	7.80 (3.52–13.16)	.136
MedianCD34 + count, 10^6^/kg (range)	5.65 (1.15–15.16)	5.41 (0.98–16.46)	.471
Follow-up time in survivors from transplant, median (range), mouth	43.4 (20.5–71.5)	41.6 (18.2–72.7)	.814

*AML* acute myelogenous leukemia, *ALL* acute lymphoblastic leukemia, *ALAL* acute leukemia of ambiguous lineage, *MNC* mononuclear cell, *PBSC* peripheral blood stem cell, *BM* bone marrow

### Engraftment and disease response

Analyses of chimerism showed that 225 cases (91%) achieved full donor chimerism and 23 (9%) mixed chimerism by day + 30 post-transplantation except for two patients who died of infection (1 in HID and 1 in MSD group) and one of primary graft failure in HID group. The median time of neutrophil recovery was 13 (range, 9–48) and 12 (range, 9–41) days in HID and MSD groups (*p* = .096), respectively. The median time of platelet engraftment was 18 (range, 10–90) and 17 (range, 9–70) days, respectively, in two groups (*p* = .131).

The count of BM blasts was analyzed on day 0 to testify disease response from sequential intensified conditioning. The median percentage of BM blasts decreased from 32% (range, 7.0–98.0%) pre-conditioning to 3.0% (range, 0.0–19.0%) on day 0. The percentage of BM blasts pre-conditioning and on day 0 was similar between two groups (*p* = .602; *p* = .563, respectively). On day 30 post-transplantation, 94% of the patients achieved CR and 6% NR, and there was no difference in CR rate between two groups (94% vs 93%; *p* = .802).

### DLI for preventing relapse

According to the DLI strategy mentioned above, a total of 199 patients received DLI for preventing relapse at a median time of 99 (range 60–640) days post-transplants, including 91 (76%) cases in HID group and 108 (82%) cases in MSD group (*p* = .350) (Fig. 1**)**. Of them, 139 patients received prophylactic DLI (HID, *n* = 68; MSD, *n* = 71) and 72 preemptive DLI (HID, *n* = 28; MSD, *n* = 44). The median number of DLI was 1 (range 1–4) per patient, with 1 (range 1–4) in each group, respectively (*p* = .087)).The median count of CD3^+^ T cells pDLI was 2.8 (range, 1.1–6.2) × 10^7^/kg in HID group and 3.0 (range, 1.0–6.5) × 10^7^/kg in MSD group, respectively (*p* = .625). Until the last follow-up, 114 episodes of post-MRD+ were recorded in 92 patients including 60 cases before DLI and 12 after DLI. Of them, 18 patients experienced two, and 2 had three episodes of post-MRD+. The incidence of the first episode of post-MRD+ was 28% (95% CI, 24–32) and 45% (95% CI, 40–49; *p* = .006) in HID and MSD groups, respectively.

### GVHD

The 1-year cumulative incidences of grades II–IV aGVHD were 62% (95% CI, 58–67) and 54% (95% CI, 50–58; *p* = .025), and III–IV aGVHD post-transplants were 16% (95% CI, 13–19) and 11% (95% CI, 8–13; *p* = .180) in HID and MSD groups, respectively. Of 199 patients received DLI, 70 (35%) patients developed aGVHD, including 28 (31%) in HID group and 42 (39%) in MSD group (*p* = .238). Among the 70 aGVHD, 48 (69%) involved skin, 29 (41%) liver, and 24 (34%) gastrointestinal tract. Eventually, 9 patients died of aGVHD in HID group and 5 in MSD group (*p* = .271). The 3-year cumulative incidences of cGVHD were 55% (95% CI, 50–59) and 55% (95% CI, 51–60; *p* = .789), and extensive cGHVD were 21% (95% CI, 17–25) and 19% (95% CI, 16–22; *p* = .830) in HID and MSD groups, respectively. Seventy-seven (39%) patients developed cGVHD after DLI, including 32 (35%) in HID group and 45 (42%) in MSD group (*p* = .383). Totally, 18 cases were diagnosed with bronchiolitis obliterans, including 6 cases in HID and 12 in MSD groups respectively (*p* = .232). Four patients died of cGVHD in HID group and 3 in MSD group (*p* = .711) (Table 3).

Risk factors for aGVHD included age, gender, donor type, and DLI. For cGVHD, the above variables as well as aGVHD were included. Multivariate analysis showed that HID and the number of DLI ≥ 2 were risk factors for aGVHD (*p =* .010, HR = 1.545; *p =* .029, HR = 1.463, respectively). DLI was a risk factor for cGVHD in multivariate analysis (*p* < .001, HR = 2.603).

### Relapse

**Fig 2:**
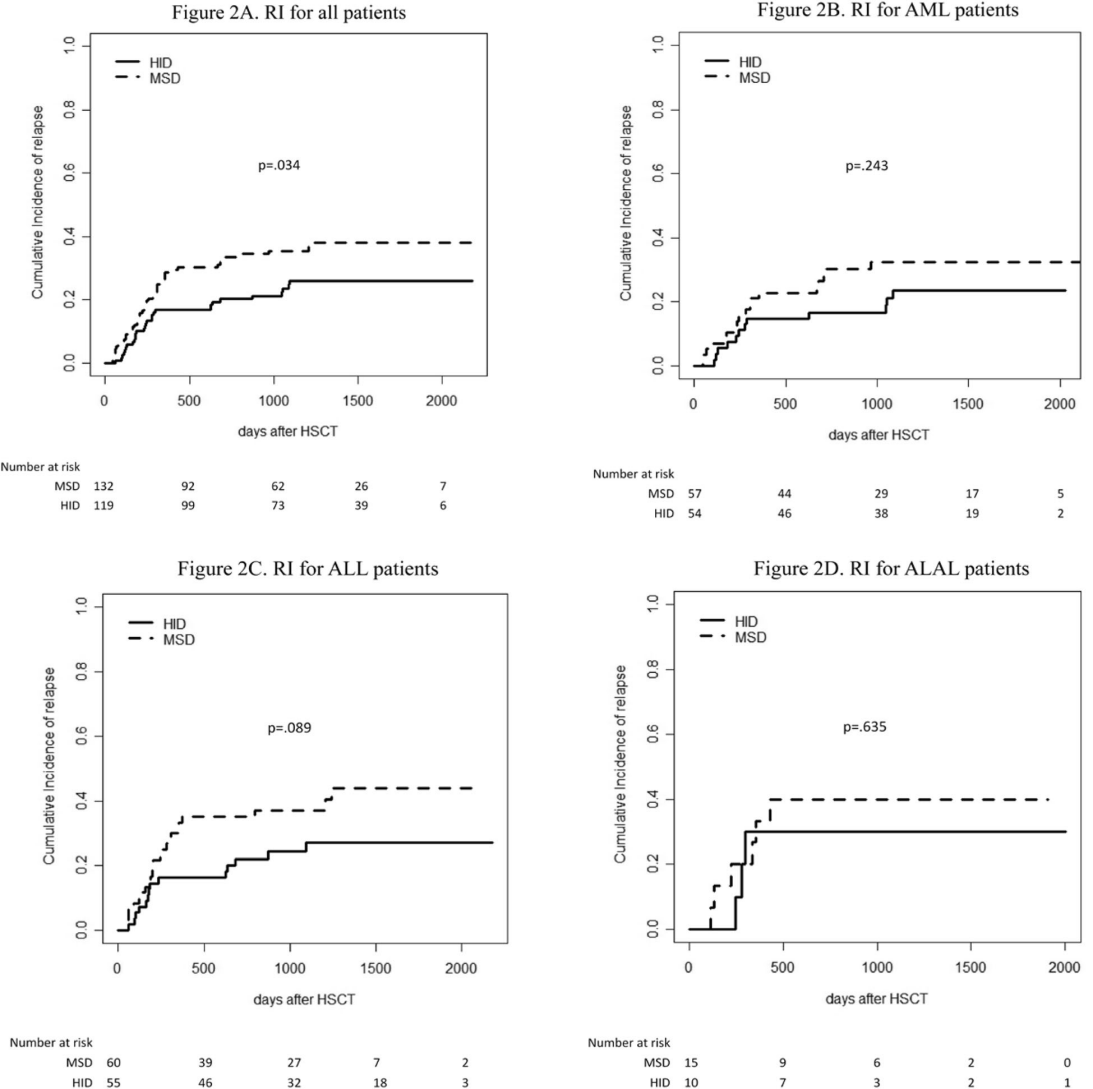
Relapse incidence after transplantation for all patients (**a**), AML patients (**b**), ALL patients (**c**), and ALAL patients (**d**)

The 5-year cumulative incidence of relapse post-transplant for all patients was 32% (95% CI, 23–41), with 26% (95% CI, 18–35) in HID group and 38% (95% CI, 29–47; *p* = .034) in MSD group (Fig. 2a), respectively. The median time of relapse was 242 (range 62–1093) and 239 (range 46–1242) days post-transplantation (*p =* .632) in HID and MSD groups, respectively.

Subgroup analysis revealed that 5-year cumulative incidence of relapse for AML,ALL, and ALAL was 23% (95% CI, 13–36), 27% (95% CI, 18–41), and 30% (95% CI, 6–60) in HID group, respectively, and 32% (95% CI, 20–45), 44% (95% CI, 29–58), and 40% (95% CI, 15–64) in MSD group, respectively (Fig. 2b for AML, *p* = .243; Fig. 2c for ALL, *p* = .089; Fig. 2d for ALAL, *p* = .635).

Of the 199 patients who received DLI, 56 relapsed, including 20 in HID and 36 in MSD groups, respectively. Of 127 patients received prophylactic DLI, 32 (25%) relapsed. Of the 60 patients received preemptive DLI, 19 (32%) relapsed. Of the 12 patients received both prophylactic and preemptive DLI, 5 (42%) relapsed. In DLI subgroup (*n* = 199), the cumulative incidence of relapse was 30% (24% in HID and 35% in MSD groups, respectively (*p* = .065)). In non-DLI subgroup (*n* = 52), 21 cases relapsed. Eight post-MRD+ cases with active GVHD received preemptive treatment of decitabine, and 4 patients relapsed. The cumulative incidence of relapse of this subgroup was 43% (33% in HID and 57% in MSD groups, respectively (*p* = .107)).

Of the 77 relapsed patients, 27 received DLI plus chemotherapy, 20 chemotherapy alone, 4 seconds allo-HSCT, and 26 abandoned further therapy. Of the 51 patients underwent treatments, 18 achieved the second CR, and 7 are still alive. Univariate analysis showed that relapse refractory, the percentage of BM blasts ≥ 3% on day 0 and post-MRD+, was an adverse factor for relapse (*p =* .048, HR = 1.448; *p =* .004, HR = 1.941; *p =* .002, HR = 1.996, respectively). In multivariate analysis, the percentage of BM blasts ≥ 3% on day 0 and post-MRD+ was an adverse factor for relapse (*p =* .037, HR = 1.652; *p =* .003, HR = 2.019, respectively). HID, DLI, and cGVHD were protective factors for relapse (*p =* .047, HR = 0.615; *p* = .034, HR = 0.561; *p =* .023, HR = 0.580, respectively) **(**Table 2**).**

**Table 2:** Univariate and multivariate analysis for relapse, DFS, and OS

Variable		Relapse		DFS		OS	
		Univariate	Multivariate (HR)	Univariate	Multivariate (HR)	Univariate	Multivariate (HR)
Donortype	HID vs MSD	***P*= .034**	***P*= .047 (0.615)** **95%CI 0.381–0.994**	***P*= .665**	**–**	***P*= .832**	**–**
Male/female		***P*= .362**	**–**	***P*= .680**	**–**	***P*= .433**	**–**
Patient age, ≥ 28 years old vs < 28 years old		***P*= .183**	**–**	***P*= .219**	**–**	***P*= .376**	**–**
AML/non-AML		***P*= .204**	**–**	***P*= .518**	**–**	***P*= .448**	**–**
Genetics:poor-risk vs others		***P*= .321**	**–**	***P*= .254**	**–**	***P*= .489**	**–**
Disease stage at transplants:relapse refractory vs PIF		***P*= .048**	***P*= .121 (1.449)** **95%CI 0.907–2.314**	***P*= .084**	***P*= .148 (1.288)** **95%CI 0.914–1.815**	***P*= .131**	**–**
WBC count at diagnosis:high vs others		***P*= .659**	**–**	***P*= .343**	**–**	***P*= .247**	**–**
BM blasts before conditioning, ≥ 32% vs < 32% (median)		***P*= .191**	**–**	***P*= .206**	**–**	***P*= .245**	**–**
BM blasts on day 0, ≥ 3% vs < 3% (median)		***P*= .004**	***P*= .037 (1.652)** **95%CI 1.032-2.640**	***P*< .001**	***P*= .005 (1.630)** **95%CI 1.162-2.288**	***P*= .001**	***P*= .011 (1.573)** **95%CI 1.110-2.229**
MRD status post-transplant, pos vs neg		***P*= .002**	**P = .003 (2.019)** **95%CI 1.267–3.216**	***P*= .005**	***P*= .003 (1.668)** **95%CI 1.185–2.347**	***P*= .019**	***P*= .027 (1.490)** **95%CI 1.048–2.121**
DLI vs no DLI		***P*= .025**	***P*= .034 (0.561)** **95%CI 0.329–0.957**	***P*< .001**	***P*< .001 (0.402)** **95%CI 0.275–0.588**	***P*< .001**	***P*< .001 (0.423)** **95%CI 0.289–0.620**
II-IV aGVHD vs 0-I aGVHD		***P*= .212**	**–**	***P*= .530**	**–**	***P*= .558**	**–**
cGVHD vs no cGVHD		***P*= .003**	***P*= .023 (0.580)** **95%CI 0.363–0.928**	***P*= .003**	***P*= .016 (0.659)** **95%CI 0.470–0.925**	***P*= .050**	***P*= .108 (0.788)** **95%CI 0.557–1.116**

*HID* haploidentical related donor, *MSD* matched sibling donor, *AML* acute myelogenous leukemia, *non-AML* acute leukemia other than AML, *OS* overall survival, *DFS* disease free survival, *PIF* primary induction failure, *BM* bone marrow, high *WBC count at diagnosis* WBC count ≥ 30,000 per mm^3^, MRD minimal residual disease

### Infections

In total, 41 patients died of infections, including 22 in HID group and 19 in MSD group, and the overall incidences of infection-related mortality were 19% (95% CI, 15–22) and 14% (95% CI,11–18), respectively, with no difference between two groups (*p* = .352). However, within the first 100 days’ post-transplant, there was higher infection-related mortality in HID than MSD (8% vs 2%, *p* = .049).

The 1-year cumulative incidence of EBV-emia was 46% (95% CI, 42–51) and 17% (95% CI, 14–21; *p* < .001) in HID and MSD groups, respectively. The 2-year cumulative incidence of EBV-associated diseases was 14% (95% CI, 11–18) and 6% (95% CI, 4–8; *p* = .027) in two groups, respectively. Ten died of EBV-associated diseases, including 6 in HID group and 4 cases in MSD group (5% vs 3%; *p* = .524). The 1-year cumulative incidence of CMV-emia was 66% (95% CI, 62–71) and 41% (95% CI, 37-45; *p* < .001) in two groups, respectively. The 2-year cumulative incidence of CMV-associated diseases was 6% (95% CI, 4–8) and 6% (95% CI, 4–8; *p* = .977) in two groups, respectively. Five died of CMV-associated diseases, including 3 in HID group and 2 cases in MSD group (3% vs 2%; *p* = .670).

### Survival

**Fig 3:**
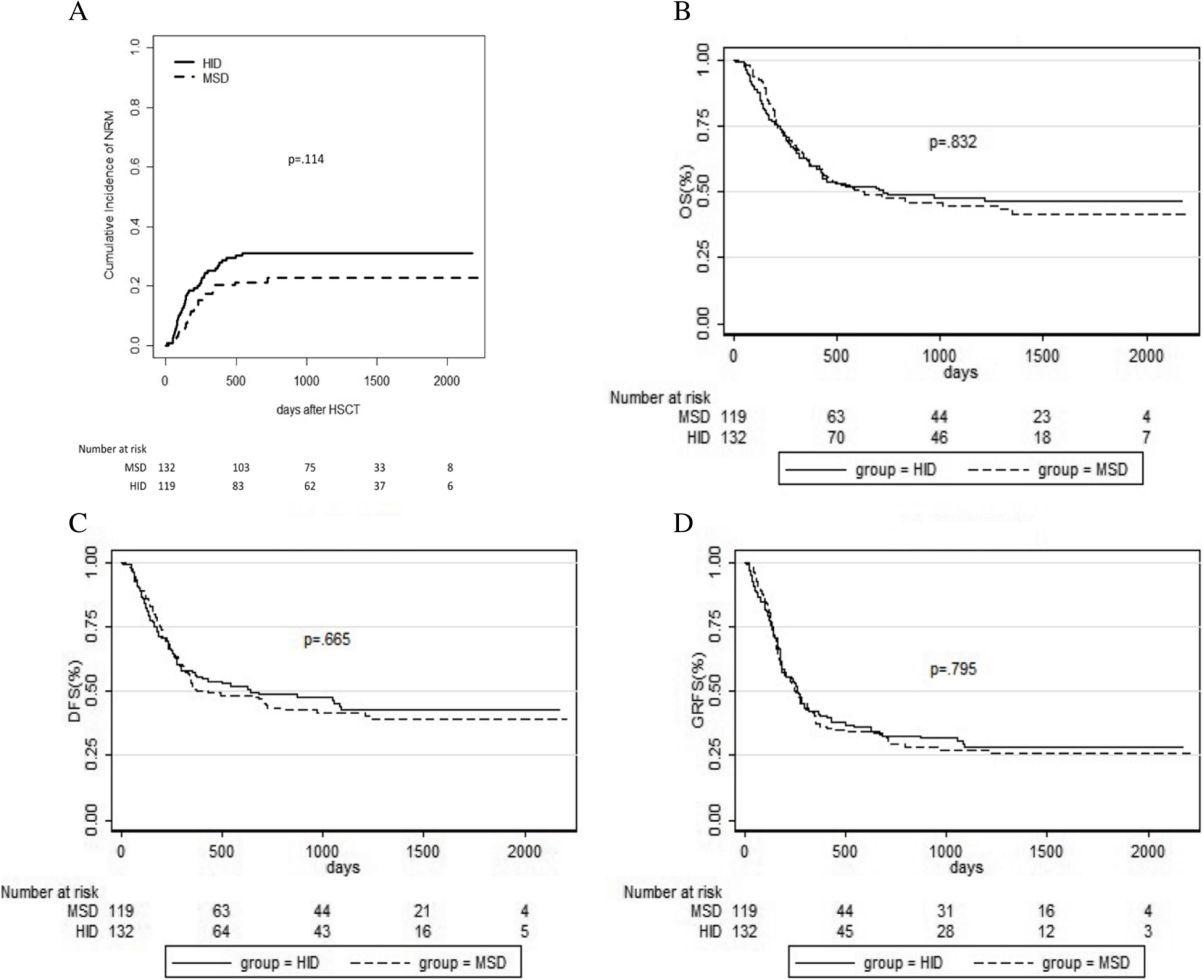
Transplant outcomes including NRM (**a**), OS (**b**), DFS (**c**), and GRFS (**d)**

At a median follow-up of 20.3 (range, 0.2–72.7) months post-transplantation, 114 patients survived, and 137 died (63 in HID and 74 in MSD). The causes of death included relapse (*n* = 69), infectious diseases (*n* = 41), GVHD (*n* = 21), sinusoidal obstruction syndrome (*n* = 1), thrombotic microangiopathy (*n* = 1), multiple organs failure (*n* = 2), primary graft failure (*n* = 1), and unknown (*n* = 1) (Table 3). The 5-year cumulative incidence of NRM was 31% (95% CI, 22–40) and 23% (95% CI,16–30; *p* = .114) **(**Fig. 3a) in HID and MSD groups, respectively. The 5-year OS was 46% (95% CI, 42–51) and 42% (95% CI,37–46; *p* = .832) **(**Fig. 3b), DFS was 43% (95% CI, 38–48) and 39% (95% CI,35–44; *p* = .665) **(**Fig. 3c), and GRFS was 28% (95% CI, 24–33) and 26% (95% CI,22–30; *p* = .795) **(**Fig. 3d) in HID and MSD groups, respectively.

**Table 3:** Causes of death post-transplantation

Cause of death	HID group	MSD group	*p*
	no. (%)	no. (%)	
Relapse	26 (21.8)	43 (32.6)	.057
aGVHD	9 (7.6)	5 (3.8)	.271
cGVHD	4 (5.9)	3 (3.7)	.711
Infections	22 (18.5)	19 (14.4)	.398
others	2 (1.7)	4 (3.0)	.686

Subgroup analysis showed that 5-year OS for AML, ALL, and ALAL was 50% (95% CI, 43–57), 43% (95% CI, 36–51), and 40% (95% CI, 27–53) in HID group, respectively, and 44% (95% CI, 38–51), 38% (95% CI, 31–46), and 40% (95% CI, 25–56) in MSD group, respectively, and there was no difference between two groups for each underlying disease (*p* = .947 for AML; *p* = .798 for ALL; *p* = .927 for ALAL).

Multivariate analysis showed that DLI was a protective factor for OS and DFS (*p <* .001, HR = 0.423; *p <* .001, HR = 0.402, respectively), and cGVHD was associated with better DFS (*p =* .016, HR = 0.659).The percentage of BM blasts ≥ 3% on day 0 and post-MRD+ was an adverse factor for OS (*p =* .011, HR = 1.573; *p =* .027, HR = 1.490, respectively) and DFS (*p =* .005, HR = 1.630; *p =* .003, HR = 1.668, respectively) (Table 2).

## Discussion

For the patients with refractory acute leukemia, allo-HSCT is the optimal curative treatment, which may allow 17–53% of patients to achieve long-term survival [7, 9, 10, 34]. Fascinatingly, a promising strategy of salvage chemotherapy with sequential conditioning was reported by our and other transplant centers, with a chance of 30–53% 3-year or 5-year OS [7, 10, 35, 36]. In this study, we obtained similar survival results [7, 10]. For patients lacking an MSD or suitably unrelated donor, HID can be an alternative choice because of its rapid and easy availability. A recent large sample data from EBMT registry [16] revealed that HID transplant had similar RI, higher NRM, and inferior survival rates than MSD transplant for refractory/relapse acute leukemia. Compared with the study of EBMT, our result showed that HID transplant had lower RI compared with MSD transplant in refractory acute leukemia. The result was consistent with our historical studies [18, 21] and other studies [17, 19], which had lower RI in HID for high-risk leukemia. In this study, our results showed that HID transplant also had a higher incidence of NRM compared with MSD, but it did not show a statistical difference between two groups. Though there was significantly lower RI in HID than MSD, the incidences of OS and DFS was similar between two groups. The reasonable explanation for these results might be the advantage of lower RI in HID group that was offset by relatively higher NRM.

Relapse is a major cause of treatment failure after allo-HSCT, especially for refractory leukemia. Many factors might influence relapse, such as transplant strategy, underlying diseases, donor resources, genetics, and disease status at transplantation [7, 16, 22, 37]. The data of EBMT showed that 2-year relapse rates for refractory/relapse leukemia were 50% in haplotransplant and 51% in MSD transplant, respectively [16]. Our result showed that the 5-year cumulative incidence of relapse was 32% in this population of refractory acute leukemia, with 25% in HID and 38% in MSD transplants. Compared with outcomes of EBMT, ours were better regardless of HID or MSD. The favorable outcomes might be attributed to the following two aspects: sequential intensified conditioning greatly decreased the leukemia burden and early tapering of immunosuppressant combined with DLI further enhanced anti-leukemia effect [7, 10, 35, 38]. Some studies suggested that AML had lower RI and better OS than ALL for refractory or advanced-stage diseases [39–41] and this study as well as our previous report [10] showed that AML and ALL had comparable RI and OS, suggesting the strategy of sequential intensified conditioning followed by DLI might be equally beneficial for both diseases. For donor resources, some studies showed that HID had stronger GVL than MSD, making relapse lower [17–19, 22, 42]. Conversely, some other studies found no advantage of GVL in haplo setting [13, 16, 43]. Our results showed that HID was associated with lower incidence of post-MRD+ and hematologic relapse compared with MSD and proved to be a protective factor in multivariate analysis of relapse, suggesting a superior GVL for refractory acute leukemia. In subgroup analysis, HID had a lower relapse tendency than MSD for ALL (*p* = .089). Poor genetics was not a related factor to relapse, which might be explained by that all patients enrolled in this study were diagnosed with refractory leukemia, and most of them were poor-risk in genetics. Higher percentage of BM blasts on day 0 post-transplantation was an adverse factor for relapse, as we reported previously [10]. Besides, Schmid et al. observed that it is more effective for PIF than relapse refractory patients by using the strategy of sequential conditioning followed by DLI [35, 38].Our results showed that patients with relapse refractory diseases had a higher risk of relapse than PIF in univariate analysis, but not in multivariate analysis.

Two main causes of NRM included GVHD and infections. In this study, a T cell replete protocol by using ATG for GVHD prophylaxis was implemented in HID transplant. Our results showed that after excluding the effect of DLI, grades II–IV aGVHD were higher for HID than MSD, but severe aGVHD and cGVHD were comparable between two groups, consistent with our data previously [22, 44, 45]. In order to induce GVL, a strategy of Immunosupressant withdrawal and DLI was used. The two methods both face the risk of GVHD. In this study, although the incidences of GVHD increased in both HID and MSD settings, the mortality of GVHD was acceptable, which was attributed to the effective interventions of DLI-related GVHD [10, 39, 46]. Meanwhile, there was no difference in terms of both aGVHD and cGVHD between two groups, consistent with our historical reports [44, 45].

Infection-related mortality was a major barrier to success of transplant. Some studies found that sequential intensified conditioning and ATG were associated with high incidence of infections, especially in the early period post-transplantation [24, 25, 47, 48]. Recently, our multicenter randomized study found that sequential intensified conditioning along with ATG for GVHD prophylaxis was positively associated with viral infections [24]. In the current study, despite no difference in overall infection-related mortality, HID transplant had higher infection-related deaths than MSD transplant within 100 day post-transplantation, coherent with our previous result [24].

The major limitation of this study was that it is a retrospective analysis of two prospective data. Therefore, further prospective multicenter studies are needed to validate our findings.

## Conclusions

In conclusion, HID transplant has lower relapse, but higher infection-related mortality and similar survival rates in refractory acute leukemia by the strategy of sequential intensified conditioning followed by DLI compared with MSD transplant.

## Data Availability

All data generated or analyzed during this study are included in this published article.
